# Effect of air pollution on household insurance purchases. Evidence from China household finance survey data

**DOI:** 10.1371/journal.pone.0242282

**Published:** 2020-11-12

**Authors:** Wenxia Zhao

**Affiliations:** Institute of Resources, Environment and Ecology, Tianjin Academy of Social Sciences, Tianjin, China; Institute for Advanced Sustainability Studies, GERMANY

## Abstract

In recent years, the health and economic effects of air pollution have attracted considerable attention, and health and insurance services have been closely related to residents’ welfare. However, there are few studies on the influence of pollution on household purchases of insurance. Using data from the 2013 and 2015 China Household Finance Surveys, this study investigates the effect of air pollution on insurance purchases using Logit and Poisson regression models. It is found that air pollution significantly increases the probability of household insurance purchases and the level of premium expenditure, although the impact of air pollution on insurance purchases shows a degree of heterogeneity. Health insurance is more sensitive to air pollution than life insurance and other types of insurance. In areas where NO_2_ and O_3_ are the main types of pollutants, air pollution has a greater impact on household insurance purchases.

## Introduction

People’s lives are closely related to environmental problems. Environmental quality not only affects the health of residents, but also has a significant impact on people’s behavior and decision-making. In recent years, countries all over the world, especially developing countries such as China, have been plagued by environmental pollution. Thus, whether household insurance decision-making changes due to environmental pollution is an important research topic.

This study investigates the effect of air pollution on household insurance purchases. Using 2013 and 2015 China Household Finance Survey (CHFS) data, which are collected by the Survey and Research Center for China Household Finance of Southwest University of Finance and Economics, we examine the influence of environmental pollution on household insurance purchases. The results show that air pollution significantly increases the probability of household insurance purchases and the level of premium expenditure, although the impact of air pollution on insurance purchases shows a degree of heterogeneity. Health insurance is more sensitive to air pollution than life insurance and other types of insurance, and different pollutants have different effects. In areas where NO_2_ and O_3_ are the main types of pollutants, air pollution has a greater impact on household insurance purchases.

Numerous epidemiological studies have shown that air pollution can damage people’s health. The health risks associated with air pollution are mainly manifested in changes in respiratory and cardiovascular functions, such as increased susceptibility to allergy [1], asthma [2, 3] and heart attacks [4]. In addition, air pollution can also cause more subtle problems, such as changes in blood pressure [5], mild headaches, and irritations of the ears, nose, and throat [4, 6]. Air pollution also affects the life expectancy of residents. Some scholars found that economic growth can reduce the mortality rate from non-respiratory infectious diseases by improving the medical environment, thereby improving the health level of residents [7]. However, air pollution, a by-product of economic growth, can increase the mortality rate from cardiopulmonary diseases and respiratory diseases, thereby shortening life expectancy. Based on the survey data of China Family Panel Studies, experts found that 16% and 9.11% of individual health status are caused by the family and community levels respectively, and the health effects of pollution is mainly reflected by its interaction with gender [8].

Air pollution affects not only the physical health of residents, but also their mental health and subjective welfare. Some recent studies matched Chinese Family Panel Studies (CFPS) data with daily average air pollution data using visit dates and locations to study the effects of air pollution on subjective well-being across multiple dimensions [9]. The results showed that air pollution can significantly reduce people’s hedonic well-being and increase their tendency toward depression, although it had no significant effect on life satisfaction. Using CFPS 2014 data, another study found that each additional standard deviation in air pollution increased the severity of mental illness by 0.38 standard deviations, resulting in a 6.67% increase in the incidence of severe mental illness [10].

Individuals frequently engage in behaviors aimed at reducing the exposure to air pollution, though these actions have associated costs. A few studies used survey data to assess how the defensive behaviors vary over multiple types of environmental risk [11]. Several experts examine the immediate effect of air pollution on a housing purchase, and their main result suggests that the transaction prices on a severely polluted day are 0.65% higher than those of the days without pollution [12]. In another study, it is found that there was a certain negative correlation between air pollution and medical insurance: the higher the degree of air pollution, the worse the self-rated health, and the fewer opportunities there are to purchase medical insurance [13].

Few studies have examined the impact of environmental pollution on residents’ or households’ insurance behavior. A study established that people’s insurance purchasing behavior displayed high degrees of randomness and irrationality based on transaction data from one insurance company in China. For every standard deviation increase in the urban air pollution index, the number of insurance contracts sold that day increased by 7.2%, while on days when the urban air pollution index was one standard deviation lower than on the day of insurance purchase, the number of cancelled insurance policies increased by 4.0% [14]. Based on the China Health and Nutrition Survey (CHNS) data, some scholars argue that medical insurance significantly promotes household durable goods consumption [15].

Some symptoms associated with air pollution may occur immediately, such as eyes or throat irritation, while other symptoms may not occur until several days or even years later, such as impacts on life expectancy and mental health. These short- and long-term impacts have an imperceptible effect on people’s expectations, and consequently affect household insurance decision-making behavior. However, there have been few studies on the impact of air pollution on household insurance purchases and premium expenditure. Insurance is one of the largest industries in the world, after real estate, finance, and government services [14]. In 2016, the consumption of health-care goods and services accounted for an average of 8.9% of GDP in OECD countries, while in the United States, the figure was 17.2%. In 2017, average per capita medical expenditure in OECD countries was about $4069, which was about 70% of average per capita education expenditure. In the United Kingdom, Iceland, Denmark and Sweden, around 80% of all spending is financed by national or regional government schemes, such as the National Health Service, while in Germany, France, and Japan on the other hand, 70% or more of health-care costs are covered by social health insurance(http://www.oecd.org/els/health-systems/health-data.htm). Understanding the impact of air pollution on household insurance purchases helps us to understand the real economic cost of air pollution, and also provides a valuable reference point for the formulation and implementation of air pollution prevention and control policies.

Generally speaking, the impact of air pollution on household insurance purchases has been discussed in a meaningful way. However, it has not been analyzed using micro-data, and thus this study will help us to understand the real economic burden of air pollution on households. It will also help local governments to formulate targeted environmental protection measures based on regional differences.

The contributions of this study are as follows. First, the impact of air pollution on household decision-making behavior in relation to insurance is estimated using micro-data. Air quality affects not only residents’ health, but also their insurance demand and expenditure level. The latter is the real economic cost borne by residents, which is seldom discussed in the existing literature. This study enriches the literature on environmental issues, focusing on the impact of air pollution on household insurance purchases from a new perspective. Second, the impact of air pollution on different types of insurance purchases differs. This study uses micro-data to analyze these differences in detail. Finally, this paper exploits a comprehensive identification of environment pollution at a city level and investigate the heterogeneity effects of pollutant sources on household insurance purchases.

## Empirical strategy

### Data

The micro-data used in this study are derived from the CHFS, which is conducted by the Survey and Research Center for China Household Finance of Southwest University of Finance and Economics [16, 17]. The CHFS uses a three-stage, stratified, proportional to population size (PPS) sampling method. Using scientific sampling and modern survey technology and survey management methods, it collects micro-information on Chinese households’ finance and provides high-quality micro-data enabling domestic and foreign researchers to study China’s household finance issues. It is also one of the most comprehensive databases in terms of household insurance-related information. In 2010, 2013, and 2015, there were 8 438, 28 141, and 37 289 households sampled, respectively. The 2013 survey covered 29 provinces, 363 counties, and 1 439 village (residential) committees throughout China with the exception of Tibet, Xinjiang, Hong Kong, Macao, and Taiwan. Because the questionnaires in 2013 and 2015 were well-matched, we used the data sets from the 2013 and 2015 surveys to build a panel data set.

### Dependent variables

There are two main ways to measure the dependent variables. One is to measure whether the respondents have insurance (including insurance purchased overseas), while the other is to measure the premium expenditure of the respondents over the preceding year. The insurance options included in the CHFS 2013 questionnaire include life insurance, health insurance, endowment insurance, property insurance, and other insurance, while the CHFS 2015 questionnaire only included life insurance, health insurance and other insurance. In terms of variable construction, based on the classifications used in CHFS 2015, if no one in the family has any insurance cover, the insurance purchase variable is 0, otherwise it is 1. The CHFS questionnaires ask family members how much they paid in premiums during the preceding year, and the responses can be used to calculate the household’s insurance premium expenditure. Because the premium expenditure reported in CHFS 2013 was the amount spent on premiums in 2012, after considering the range of air quality variables used in this study, data from the 2015 questionnaire were used in the premium expenditure model.

### Independent variables

PM2.5 concentration data have been used to measure air quality in numerous recent studies [10], but PM2.5 is only one of several main types of air pollution, and thus it is more appropriate to consider the air quality indicators related to various types of pollutants as a proxy for the level of air pollution. Considering the different types of pollutants in different cities, to facilitate comparisons, we used a comprehensive air quality index to measure air pollution in different locations. The comprehensive air quality index takes into account the degree of pollution in terms of SO_2_, NO_2_, PM10, PM2.5, CO, and O_3_. The larger the value, the heavier the degree of pollution. Because the CHFS did not publish data for the cities where the respondents were located, considering that the population in the provinces in China is concentrated in the key cities, the average air quality data values for the key cities in the provinces were used as proxies for the air quality of the provinces. Since 2013, the Ministry of Environmental Protection of China has published the comprehensive air quality index values for 74 key cities. In 2015, the comprehensive air quality index values for key cities began to be published at the provincial level. The data were obtained from the websites of the Ministry of Environmental Protection of China and the provincial environmental protection bureaus. The comprehensive air quality index data are monthly data, and so the annual air quality variable was calculated using the average of the monthly data for each city. There are some missing monthly data for Jiangxi, Shandong, Shaanxi and Heilongjiang, and thus the averages of the available monthly data are used.

In addition to the above variables, we also control for the characteristics of individuals and families by referring to the existing literature. These characteristics include gender, age, marital status, education level, level of attention to economic and financial information, total family income, location (urban or rural), family size, number of children under 14 years old, possession of social endowment insurance, and possession of social medical insurance. In addition, the fixed effects of time and region are controlled for. To eliminate heteroscedasticity, both income and insurance premium expenditure are estimated in logarithmic form. Considering that the income and insurance premium expenditure of some survey respondents were zero, the logarithm of all income and insurance premium expenditure values was used after adding 1 to them.

## Methods

To test the impact of air pollution on household insurance purchases, the following model is constructed:

$$I_{ijt}=\alpha_{0}+\alpha_{1}\cdot envir_{jt}+X_{it}\cdot \theta +\varepsilon_{ijt}$$ (1)

where *I_ijt_* represents whether family *i* has insurance, which is binary, or the amount that family *i* spends on insurance premiums, which is continuous, in province *j* in year *t*. In the CHFS questionnaire, respondents were asked what kind of insurance they had. If no one in the family had life insurance, health insurance, or other insurance, the family insurance variable took a value of 0, otherwise 1. *envir* denotes the air pollution level. The vector *X_it_* incorporates other variables that may influence the dependent variable (gender, age, marital status, educational level of the head of the household, level of attention to economic and financial information, total family income, location (urban or rural), family size, number of children under 14 years old, possession of social endowment insurance, and possession of social medical insurance). *ε_ijt_* denotes the error term. Table 1 shows the summary statistics for the variables.

**Table 1:** Descriptive statistics for the variables.

Variable	Description	Mean	Standard deviation	Min	Max	Obs.
Insurance purchases	If no insurance was purchased by the household, the variable is 0, otherwise 1.	0.2413	0.4279	0	1	58915
Premium expenditure	Premium paid last year	1.0973	2.7854	0	14.5211	58915
Air quality	Air quality comprehensive index	4.9636	1.2746	1.9342	7.5296	58217
Gender	1 for male, 0 for female	0.7648	0.4241	0	1	59061
Age	Respondents’ age	52.8845	14.1764	1	113	59052
Marital	1 if the head is Married or cohabiting, 0 otherwise.	0.8668	0.3398	0	1	59023
Education	The higher the value, the higher the educational level.	3.3852	1.6645	1	9	58998
Income	Disposable income in the last year	10.4381	1.4240	0	15.4250	57081
Rural	1 if the family live in rural areas, 0 othersize	0.3332	0.4714	0	1	59061
Family size	Number of household population	3.5728	1.6893	1	20	59061
Kids number	Number of children under 14	0.5103	0.7806	0	10	59061
Attention to finance	The lower the value, the greater attention paid to finance	3.8905	1.1017	1	5	58980
Social endowment insurance	0 if the head had no such insurance, 1 othersize	0.7752	0.4175	0	1	57063
Social medical insurance	0 if the head had no such insurance, 1 othersize	0.9159	0.2776	0	1	56907

## Results

### Preliminary results

Columns (1) and (4) in Table 2 show that air quality has a significant positive impact on household insurance purchases and premium expenditure. Considering that insurance purchases is a dummy variable, column (2) uses a logit model to estimate the impact of air quality on insurance purchases. The results show that every additional unit of the comprehensive air quality index will increase the probability of households purchasing insurance by 17.5% (e^0.1615^–1). After including the variables for social endowment insurance and social medical insurance, column (3) shows that the coefficient of the air quality index is still positive and significant at the 1% level. Despite its appealing simplicity, the OLS method suffers from an important drawback when analyzing premium expenditure in that it ignores the non-negative expenditure constraint. Previous studies have addressed this issue using a Tobit model. However, the Tobit model yields inconsistent estimators in the presence of fixed effects and when the idiosyncratic error term is heteroskedastic. Partly because of these issues, we use a Poisson model. The results are presented in columns (5) and (6) in Table 2, and indicate that each additional unit of the comprehensive air quality index increases household expenditure on insurance premiums by about 7%. The premium expenditure variable is based on premiums paid during the previous year, and because air quality data are unavailable for 2012, the data used for the premium expenditure estimations are from 2015.

**Table 2:** Impact of air pollution on family insurance purchases and premium expenditure.

Variables	(1)Insurance Purchase (OLS)	(2)Insurance Purchase (Logit)	(3)Insurance Purchase (Logit)	(4)Premium Expenditure (OLS)	(5)Premium Expenditure (Possion)	(6)Premium Expenditure (Possion)
Air quality	0.0075(0.0016)***	0.1615(0.0101)***	0.0526(0.0120)***	0.0620(0.0117)***	0.0668(0.0106)***	0.0626(0.0107)***
Gender	-0.0284(0.0048)***	-0.2036(0.0338)***	-0.2025(0.0361)***	-0.2098(0.0377)***	-0.1287(0.0303)***	-0.1249(0.0305)***
Age	-0.0013(0.0001)***	-0.0108(0.0010)***	-0.0139(0.0011)***	-0.0145(0.0010)***	-0.0161(0.0010)***	-0.0173(0.0011)***
Marital	-0.0442(0.0057)***	-0.3019(0.0433)***	-0.3317(0.0468)***	0.3079(0.0399)***	0.4259(0.0526)***	0.4050(0.0534)***
Education	0.0284(0.0015)***	0.1967(0.0100)***	0.1981(0.0109)***	0.1541(0.0116)***	0.0859(0.0089)***	0.0800(0.0092)***
Income	0.0107(0.0014)***	0.0921(0.0109)***	0.0745(0.0115)***	0.2051(0.0103)***	0.2565(0.0143)***	0.2512(0.0146)***
Rural	-0.0283(0.0043)***	-0.2558(0.0330)***	-0.2514(0.0358)***	-0.2924(0.0295)***	-0.5205(0.0437)***	-0.5253(0.0443)***
Family size	0.0564(0.0016)***	0.4272(0.0117)***	0.4533(0.0128)***	0.0029(0.0107)	0.03240.0106)***(	0.0397(0.0107)***
Kids number	-0.0364(0.0034)***	-0.2823(0.0227)***	-0.2949(0.0246)***	0.1688(0.0251)***	0.1049(0.0222)***	0.0954(0.0226)***
Attention to finance	-0.0280(0.0017)***	-0.1845(0.0121)***	-0.2014(0.0131)***	-0.2311(0.0147)***	-0.1813(0.0120)***	-0.1816(0.0121)***
Social endowment insurance			0.1553(0.0369)***			0.0916(0.0411)**
Social medical insurance			-0.0013(0.0524)			0.0314(0.0534)
Constant	0.0140 (0.0207)	-3.6121(0.1548)***	-3.3238(0.1700)***	-0.3625(0.1611)**	-2.3302(0.1934)***	-2.2373(0.2017)***
Region FE	Y	N	Y	Y	N	Y
Year FE	Y	N	Y	N	N	N
N	55972	55972	53800	34397	34397	33875

*Notes*: In brackets are robust standard errors.

* p<0.10

** p<0.05

*** p<0.01.

When other factors remain unchanged, gender, age, and level of attention to economic and financial information for the head of household have a significant negative impact on family insurance purchases and premium expenditure. The probability of male-headed households purchasing insurance is 18.3% lower than that of female-headed households (see column (3)), and expenditure on insurance premiums is 11.7% lower than that of female-headed households (see column (6)). Education level and income level have a positive impact on household insurance purchases and premium expenditure. The larger the family, the more likely they are to buy insurance and the higher their premium expenditure compared with smaller families. Families living in rural areas are significantly less likely to buy insurance than those living in urban areas, and the premium expenditure of those that buy insurance is significantly lower than that of urban families. Families with social endowment insurance are more likely to buy insurance and pay higher premiums than those without social endowment insurance. Possession of social medical insurance has no significant impact on family insurance purchases and premium expenditure. The variables related to marriage and the number of children in the family have asymmetric effects on insurance purchases and premium expenditure. Families of married or cohabiting couples are less likely to purchase insurance than those of other households, although those that purchase insurance spend more on premiums than other types of households. Perhaps this is because the more children in the household under 14 years old, the heavier the family burden, and so these families are not highly motivated to purchase insurance, but those that do purchase insurance may spend more on premiums than families with fewer children.

### Robustness tests

First, considering that the air quality data for key cities published by the China Ministry of Environmental Protection may not accurately measure the air quality in rural areas, there may be deviations as a result of using this index. Therefore, the model was regressed while excluding those living in rural areas. The results are shown in columns (1) and (2) in Table 3 and are consistent with those shown in Table 2. Second, the effects of some special areas on the model’s estimation are eliminated. Columns (3) and (4) show the results after deleting the two provinces with the least number of observations, namely, the Inner Mongolia Autonomous Region and the Ningxia Hui Autonomous Region. The results are almost identical to those shown in Table 2, indicating that the results are robust.

**Table 3:** Results of robustness tests.

Variables	(1)Insurance Purchase (Logit)	(2)Premium Expenditure (Possion)	(3)Insurance Purchase (Logit)	(4)Premium Expenditure (Possion)	(5)Insurance Purchase (Logit)	(6)Premium Expenditure (Possion)
Air quality	0.0743(0.0153)***	0.0488(0.0114)***	0.0518(0.0122)***	0.0615(0.0107)***	0.3961(0.1716)**	0.0891(0.0428)**
Gender	-0.1612(0.0420)***	-0.1430(0.0315)***	-0.2064(0.0369)***	-0.1305(0.0308)***	-0.2005(0.1757)	-0.1267(0.0447)***
Age	-0.0162(0.0014)***	-0.0152(0.0011)***	-0.0138(0.0012)***	-0.0169(0.0011)***	-0.0246(0.0055)***	-0.0165(0.0016)***
Marital	-0.2973(0.0580)***	0.3803(0.0559)***	-0.3290(0.0478)***	0.4084(0.0543)***	-1.2452(0.2459)***	0.3693(0.0785)***
Education	0.1908(0.0128)***	0.0815(0.0096)***	0.2058(0.0111)***	0.0820(0.0093)***	0.3952(0.0558)***	0.0591(0.0134)***
Income	0.1411(0.0154)***	0.2368(0.0159)***	0.0711(0.0118)***	0.2473(0.0148)***	0.2467(0.0567)***	0.2378(0.0212)***
Rural			-0.2454(0.0367)***	-0.5235(0.0451)***	-3.2251(1.1070)***	-1.7990(0.9847)*
Family size	0.4768(0.0174)***	0.0516(0.0118)***	0.4596(0.0132)***	0.0411(0.0108)***	1.4851(0.1016)***	0.0305(0.0166)*
Kids number	-0.2398(0.0346)***	0.1113(0.0260)***	-0.2987(0.0252)***	0.0950(0.0230)***	-0.7777(0.1931)***	0.1514(0.0367)***
Attention to finance	-0.2370(0.0168)***	-0.1896(0.0132)***	-0.2049(0.0134)***	-0.1845(0.0123)***	-0.6026(0.0844)***	-0.1756(0.0187)***
Social endowment insurance	0.1917(0.0500)***	0.1127(0.0462)**	0.1878(0.0380)***	0.1042(0.0420)**	0.0960(0.2196)	0.0682(0.0660)
Social medical insurance	-0.0160(0.0627)	-0.0188(0.0559)	0.0046(0.0536)	0.0367(0.0545)	0.5283(0.2676)**	0.0186(0.0786)
Constant	-3.9643(0.2201)***	-2.0629(0.2186)***	-3.3778(0.1741)***	-2.2216(0.2042)***	-8.1088(0.8025)***	-1.6048(0.2822)***
Region FE	Y	Y	Y	Y	Y	Y
Year FE	Y	N	Y	N	N	N
N	36120	23593	52191	32962	11013	11013

*Notes*: Robust standard errors are in parentheses.

* p<0.10

** p<0.05

*** p<0.01.

Third, a different method was used to measure air quality. In the CHFS 2015 survey, the respondents were asked how serious they thought the pollution was in their location. If they responded that air pollution in their city was serious, the variable was allocated a value of 1, otherwise 0. Because only responses for 2015 were available, the number of observations was reduced to 11 013. The results are shown in columns (5) and (6) of Table 3. It can be seen that the air quality variable is still positive and significant at the 5% level, indicating that the more serious the air pollution in the city, the more likely the respondents are to buy insurance and increase their premium expenditure. Table 3 also shows that the signs of the coefficients and the significance levels of other variables have not changed markedly, indicating that deleting some samples or changing air quality measurement methods does not change the main results of this study.

### Endogeneity tests

The problem of missing variables may still exist in view of the limitations of the data and the complexity of the correlations between variables. Non-observable variables may simultaneously affect air quality and family insurance purchases, and thus we attempted to select the appropriate instrumental variables(IV) to solve the endogeneity problem.

First, considering the regional characteristics of air quality, that is, the air quality in a given region is likely to show some degree of commonality, and thus the air quality of different cities in the same province is likely to be similar, the air quality of the city where a family lives is often similar to that of the relevant provincial capital city. However, generally, the air quality of provincial capital cities does not directly affect a family’s insurance purchases and premium expenditure. Therefore, we use the air quality of provincial capital cities as an instrumental variable. The results are shown in columns (1) and (2) of Table 4.

**Table 4:** Results of endogeneity testing.

Variables	(1)Insurance Purchase	(2)Premium Expenditure	(3)Insurance Purchase	(4)Premium Expenditure
Air quality	0.0090(0.0018)***	0.0588(0.0116)***	0.0134(0.0022)***	0.0551(0.0129)***
Gender	-0.0277(0.0045)***	-0.1978(0.0288)***	-0.0331(0.0057)***	-0.2068(0.0358)***
Age	-0.0015(0.0001)***	-0.0185(0.0009)***	-0.0008(0.0002)***	-0.0153(0.0012)***
Marital	-0.0468(0.0058)***	0.2622(0.0374)***	-0.0742(0.0074)***	0.2900(0.0461)***
Education	0.0269(0.0014)***	0.1542(0.0088)***	0.0300(0.0017)***	0.1536(0.0109)***
Income	0.0112(0.0014)***	0.2139(0.0093)***	0.0025(0.0018)	0.2046(0.0112)***
Rural	-0.0277(0.0044)***	-0.3181(0.0282)***	-0.0138(0.0058)**	-0.2984(0.0360)***
Family size	0.0589(0.0015)***	0.0010(0.0098)	0.0847(0.0019)***	0.0069(0.0107)
Kids number	-0.0397(0.0031)***	0.1639(0.0203)***	-0.0655(0.0041)***	0.1600(0.0257)***
Attention to finance	-0.0286(0.0017)***	-0.2196(0.0112)***	-0.0283(0.0023)***	-0.2300(0.0143)***
Social endowment insurance	0.0177(0.0046)***	0.0717(0.0300)**	0.0218(0.0061)***	0.0617(0.0383)
Social medical insurance	-0.0003(0.0067)	-0.0719(0.0436)*	0.0110(0.0088)	-0.0082(0.0554)
Constant	0.0128(0.0220)	0.0197(0.1421)	0.0210(0.0287)	-0.3083(0.1809)*
Region FE	Y	Y	Y	Y
Year FE	Y	Y	N	N
N	53800	53800	33875	33875

Note: Standard errors are in parentheses

* p<0.10

** p<0.05

*** p<0.01.

In addition, because of the lag in the effect of air pollution on household insurance purchases and premium expenditure, we use the 2013 air quality index as the instrumental variable for air quality in 2015. The results are shown in columns (3) and (4) of Table 4, and are basically consistent with those shown in columns (1) and (2) of Table 4.

To enable comparison with the results of the baseline regression shown in columns (1) and (4) of Table 2, two stage least square(2SLS) regression with IV was then used in columns (1) and (3) of Table 4. In the estimation, the Cragg−Donald F-statistic for the first-stage regression is greater than 10, indicating that there is no weak instrumental variable problem, and the model’s estimations regarding the effect of air pollution are robust.

## Heterogeneity analysis

### Different types of insurance

Considering the obvious differences between life insurance and health insurance, the impact of air quality on different types of insurance may also be different. In Table 5, columns (1)−(3) show the results of regression of different types of insurance purchases, while columns (4)−(6) show the results of different types of insurance premium expenditure. It can be seen that air pollution has a positive impact on both insurance purchases and premium expenditure, and is significant at the 5% level. However, health insurance purchases and premium expenditure are significantly more affected by air pollution than life insurance purchases and premium expenditure. Specifically, every unit increase in the air quality index increases the probability of purchasing health insurance by 10.71% (e^0.1017^–1), that of purchasing life insurance by 7.95% (e^0.0765^–1), and that of purchasing other types of insurance by 6.72% (e^0.0650^–1).

**Table 5:** Impact of air quality on demand for different types of insurance.

Variables	(1)Life Insurance Purchase	(2)Health Insurance Purchase	(3) Other Insurance Purchase	(4)Life Insurance Expenditure	(5)Health Insurance Expenditure	(6) Other Insurance Expenditure
Air quality	0.0765(0.0189)***	0.1071(0.0226)***	0.0650(0.0220)***	0.0393(0.0149)***	0.0950(0.0197)***	0.0548(0.0233)**
Gender	-0.1855(0.0534)***	-0.2525(0.0616)***	-0.0853(0.0618)	-0.1350(0.0427)***	-0.2180(0.0542)***	-0.0362(0.0714)
Age	-0.0222(0.0018)***	-0.0305(0.0021)***	-0.0229(0.0020)***	-0.0143(0.0014)***	-0.0203(0.0018)***	-0.0183(0.0024)***
Marital	0.4973(0.0791)***	0.4178(0.0949)***	0.3125(0.0943)***	0.4139(0.0738)***	0.4265(0.0958)***	0.3772(0.1193)***
Education	0.1801(0.0161)***	0.1719(0.0182)***	0.0526(0.0186)***	0.0943(0.0127)***	0.1111(0.0165)***	0.0516(0.0211)**
Income	0.3884(0.0232)***	0.2912(0.0275)***	0.2526(0.0272)***	0.2922(0.0204)***	0.2429(0.0271)***	0.2781(0.0336)***
Rural	-0.5404(0.0625)***	-0.7893(0.0791)***	-0.5117(0.0737)***	-0.4267(0.0610)***	-0.7641(0.0878)***	-0.5691(0.0933)***
Family size	0.0625(0.0183)***	0.0883(0.0215)***	0.0263(0.0209)	0.0393(0.0147)***	0.0436(0.0197)**	0.0325(0.0236)
Kids number	0.0516(0.0381)	0.1172(0.0442)***	0.2708(0.0412)***	0.0448(0.0323)	0.1667(0.0416)***	0.2324(0.0465)***
Attention to finance	-0.2838(0.0195)***	-0.2740(0.0232)***	-0.2021(0.0232)***	-0.2180(0.0167)***	-0.1948(0.0218)***	-0.1841(0.0274)***
Social endowment insurance	0.1091(0.0600)*	0.2702(0.0739)***	0.1904(0.0727)***	0.0637(0.0575)	0.1714(0.0770)**	0.1771(0.0927)*
Social medical insurance	0.0140(0.0798)	-0.0417(0.0947)	-0.0941 (0.0958)	0.0355(0.0746)	0.0367(0.0999)	0.0400(0.1224)
Constant	-6.7460(0.3151)***	-6.1599(0.3664)***	-5.5591(0.3727)***	-3.1974(0.2834)***	-3.3675(0.3595)***	-3.9959(0.4725)***
Region FE	Y	Y	Y	Y	Y	Y
Year FE	Y	Y	Y	-	-	-
N	53800	53800	53800	33875	33875	33875

*Notes*: Logit models were used to estimate insurance purchases in columns (1)−(3), while Poisson models were used to estimate insurance purchases in columns (4)−(6). Robust standard errors are in parentheses.

* p<0.10

** p<0.05

*** p<0.01.

### Pollutant heterogeneity

Considering that different pollutants may have different effects, to investigate the impact of heterogeneity among various types of pollution, the environmental variables were replaced by PM2.5, PM10, SO_2_, NO_2_, O_3_,_,_ and CO concentration data, which were obtained from the website of China’s Ministry of Ecology and Environment. Since data are only available for key cities, in order to get the data of provinces’ level, we average the data of city level in each province. Because the data relating to air quality in 2013 are not available, the results of Table 6 are estimated using cross-sectional data.

**Table 6:** Effects of pollutant heterogeneity.

Variables	(1)PM2.5	(2)PM10	(3)SO_2_	(4)NO_2_	(5)O_3_	(6)CO
Air quality	1.0538(4.1127)	0.7743(0.1801)***	0.2075(0.0920)**	1.8827(0.2265)***	1.9329(0.4219)***	0.5957(0.1531)***
Gender	-0.6010(4.0369)	-0.6085(0.1385)***	-0.5956(0.1388)***	-0.5757(0.1268)***	-0.6072(0.1571)***	-0.5713(0.1218)***
Age	-0.0189(0.0432)	-0.0186(0.0037)***	-0.0177(0.0037)***	-0.0199(0.0035)***	-0.0187(0.0042)***	-0.0186(0.0034)***
Marital	-1.4604(D)	-1.4479(0.2470)***	-1.4057(0.2511)***	-1.4002(0.1950)***	-1.4680(0.1748)***	-1.3460(0.1772)***
Education	0.5626(2.4691)	0.5594(0.0696)***	0.5581(0.0717)***	0.5411(0.0540)***	0.5742(0.0751)***	0.5315(0.0488)***
Income	0.0652(0.3641)	0.0755(0.0368)**	0.0745(0.0368)**	0.0554(0.0356)	0.0597(0.0487)	0.0808(0.0343)**
Rural	-0.3874(0.7373)	-0.4251(0.1148)***	-0.4285(0.1143)***	-0.3602(0.1096)***	-0.3824(0.1293)***	-0.4070(0.1066)***
Family size	1.6607(7.5881)	1.6440(0.1891)***	1.6129(0.1962)***	1.6207(0.1280)***	1.6785(D)	1.5574(0.1068)***
Kids number	-1.2461(6.1818)	-1.2370(0.1875)***	-1.2166(0.1940)***	-1.2107(0.1439)***	-1.2616(0.1491)***	-1.1783(0.1301)***
Attention to finance	-0.5314(2.5288)	-0.5285(0.0778)***	-0.5193(0.0791)***	-0.5132(0.0624)***	-0.5392(0.0821)***	-0.5024(0.0572)***
Social endowment insurance	0.4339(1.2855)	0.4274(0.1226)***	0.4358(0.1225)***	0.4526(0.1173)***	0.4597(0.1326)***	0.4053(0.1133)***
Social medical insurance	0.2503(0.6509)	0.2459(0.1703)	0.2455(0.1694)	0.2467(0.1652)	0.2307(0.1867)	0.2386(0.1607)
Constant	-13.0518(28.0586)	-11.8437(1.0219)***	-9.1258(0.6402)***	-14.8681(0.9928)***	-18.7098(2.1907)***	-8.3775(0.5013)***
Region FE	Y	Y	Y	Y	Y	Y
N	35226	35226	35226	35226	35226	35226

*Notes*: Dependent variables are all insurance purchases. Because there is a degree of collinearity, the standard deviations for marital status in column (1) and family size in column (5) have not been estimated, and are represented by (D). Robust standard errors are in parentheses. * p<0.10

** p<0.05

*** p<0.01.

It can be seen from Table 6 that the concentrations of PM10, SO_2_, NO_2_, O_3_, and CO are positively correlated with household insurance purchases, and the impact of NO_2_ and O_3_ on household insurance purchases is significantly higher than that of the other pollutants, while the PM2.5 concentration has no significant impact on household insurance purchases. Thus, household insurance purchases are more sensitive to the level of NO_2_ and O_3_ pollution than to that of other pollutants. In other words, in areas where NO_2_ and O_3_ are the main types of pollution, there is a greater probability of households purchasing insurance.

## Conclusion

Environmental problems are one of the major challenges facing governments in the new global era, while residents’ health and insurance services are closely related to the welfare of residents. However, there have been relatively few studies on the impact of environmental pollution on household insurance purchases and premium expenditure. Using logit and Poisson models, this study examined the impact of air pollution on household insurance purchases using CHFS data. It was found that air pollution significantly increased the probability of households purchasing insurance and spending more on premiums. After considering differences in air pollution between urban and rural areas, excluding samples from some special areas, and using the lag term of the air quality index for provincial capital cities as the instrumental variable, the results remained robust. It is worth noting that the impact of air pollution on household insurance purchases shows a degree of heterogeneity. Health insurance is more sensitive to air pollution than life insurance and other types of insurance. Furthermore, the impact of air pollution on household insurance purchases is more significant in areas where NO_2_ and O_3_ are the main types of pollutants.

Because of the lack of corresponding information, we sidestep the issue whether air pollution affect the holding of other types of financial product such as stocks, foreign exchange products and mutual funds. Our analysis does not shed much light on the relationship between air pollution and household portfolio choice either. Further study in these directions needs to be conducted to contribute to the literature if more detailed information is available.

## References

[1] Zhong J., Lee Y., Hsieh C., Tseng C., and Yiin L. Association between the first occurrence of allergic conjunctivitis, air pollution and weather changes in Taiwan. Atmospheric Environment. 2019; 212:90–95.

[2] Seaton A., MacNee W., Donaldson K., and Godden D. Particulate air pollution and acute health effects. Lancet. 1995; 345(8943): 176–178. 10.1016/s0140-6736(95)90173-6 7741860

[3] Dockery D.W., and Pope C.A. Acute respiratory effects of particulate air pollution. Annual Review of Public Health. 1994; 15: 107–113. 10.1146/annurev.pu.15.050194.000543 8054077

[4] Pope C. Epidemiology of fine particulate air pollution and human health: biologic Mechanisms and who’s at risk?. Environmental Health Perspectives. 2000; 108(4): 713–723. 10.1289/ehp.108-1637679 10931790 PMC1637679

[5] Auchincloss A., Roux A.D., and Dvonch J., et al Associations between recent exposure to ambient fine particulate matter and blood pressure in the Multi-ethnic Study of Atherosclerosis(MESA). Environmental Health Perspectives. 2008; 116(4): 486–491. 10.1289/ehp.10899 18414631 PMC2291007

[6] Ghio A., Kim C., and Devlin R. Concentrated ambient air particles induce mild pulmonary inflammation in healthy human volunteers. American Journal of Respiratory and Critical Care Medicine. 2000; 162(3): 918–988. 10.1164/ajrccm.162.3.9911115 10988117

[7] Lavy V., Ebenstein A., and Roth S. The impact of short term exposure to ambient air pollution on cognitive performance and human capital formation. NBER Working Paper. 2014; No.20648.

[8] Zhang Y., Jin Y., and Zhu T. The health effects of individual characteristics and environmental factors in China: Evidence from the hierarchical linear model. Journal of Cleaner Production. 2018; 194:554–563.

[9] Zhang X., Zhang X., and Chen X. Happiness in the Air: How does a Dirty Sky Affect Mental Health and Subjective Well-being?. Journal of Environmental Economics and Management. 2017; 85:81–94. 10.1016/j.jeem.2017.04.001 29081551 PMC5654562

[10] Chen, S., P. Oliva, and P. Zhang. Air Pollution and Mental health: Evidence from China. NBER Working Paper. 2018; No. 24686.

[11] Williams A.M. Understanding the micro-determinants of defensive behaviors against pollution. Ecological Economics. 2019; 163(C):42–51.

[12] Qin Y., Wu J., and Yan J. Negotiating housing deal on a polluted day: Consequences and possible explanations. Journal of Environmental Economics and Management. 2019; 94(C): 161–187.

[13] Pi T., Wu H., and Li X. Does air pollution affect health and medical insurance cost in the elderly: An empirical evidence from China. Sustainability. 2019; 11(6): 1–49.

[14] Chang T.Y., Huang W., and Wang Y. X. Something in the air: pollution and the demand for health insurance. Review of Economic Studies. 2018; 85(3):1609–1634.

[15] Cai W., Chen J., and Ding H. Medical Insurance Effects on Household Durable Goods Consumption: Evidence from China. Emerging Markets Finance and Trade. 2016; 52(2): 449–460.

[16] Gan L., and Li Y. China Rural Family Finance Development report 2014. Chengdu: southwest University of Finance and Economics Press 2014.

[17] Gan L., Yin Z., and Tan J. Investigation report of Chinese Family Finance 2014. Chengdu: southwest University of Finance and Economics Press 2015.

